# Does Maternal Smoking Increase the Risk of Congenital Heart Disease? Insights from a Single-Center Fetal Echocardiography Study

**DOI:** 10.3390/jcm15083143

**Published:** 2026-04-20

**Authors:** Akif Kavgacı, Özkan Kaya, Utku Arman Örün, Mehmet Emre Arı

**Affiliations:** Department of Pediatric Cardiology, Ankara Etlik City Hospital, 06170 Ankara, Turkey; drozkankaya@gmail.com (Ö.K.); utkuarman@hotmail.com (U.A.Ö.); memreari@yahoo.com (M.E.A.)

**Keywords:** maternal smoking, congenital heart disease, fetal echocardiography, prenatal exposure, associated extracardiac anomalies

## Abstract

**Background**: Congenital heart disease (CHD) represents a major cause of perinatal morbidity and mortality, and fetal echocardiography is essential for its early diagnosis and management. Maternal smoking has been suggested as a potential teratogenic factor affecting fetal cardiovascular development; however, findings regarding its association with CHD remain inconsistent. This study aimed to evaluate the relationship between maternal smoking during pregnancy and the risk of CHD. **Methods**: A total of 2715 pregnant women and 2784 fetuses who underwent fetal echocardiography at ≥20 weeks’ gestation between 1 January 2024 and 1 November 2025 were analyzed. Pregnancies complicated by known chromosomal or syndromic abnormalities, significant teratogenic exposure, duplicate assessments, or nonstandard examinations were excluded. Maternal smoking status during pregnancy was recorded and categorized according to daily cigarette consumption. The prevalence of CHD and the distribution of CHD subtypes were evaluated and compared according to smoking status. Fetal cardiac diagnoses were classified based on the classical morphological classification system. **Results**: A total of 2715 pregnancies (2784 fetuses) were analyzed, including 2530 fetuses in the non-smoking group and 254 in the smoking group. Congenital heart disease was detected in 12.5% of fetuses in the non-smoking group and 14.2% in the smoking group, with no statistically significant difference (*p* = 0.442). According to the classical morphological classification, the distribution of fetal echocardiographic pathologies did not differ significantly between groups (*p* = 0.607). Septal defects were the most common subtype in both groups. Although conotruncal defects were proportionally more frequent in the smoking group, this difference did not reach statistical significance. After reclassifying daily cigarette consumption into four exposure categories, no association was detected between maternal smoking and CHD risk (OR = 1.04; 95% CI: 0.86–1.26; *p* = 0.691). **Conclusion**: In this cohort referred for fetal echocardiographic evaluation, no association was detected between maternal smoking during pregnancy and the risk of congenital heart disease or alterations in CHD subtype distribution. No consistent dose–response relationship was observed. These findings suggest that no association was detected between maternal smoking exposure and CHD. Further large-scale prospective studies are needed to clarify phenotype-specific associations.

## 1. Introduction

Congenital heart disease (CHD) is estimated to have an incidence ranging from 6 to 12 per 1000 live births [1] with reported variations across studies largely attributable to differences in diagnostic methods, inclusion of minor cardiac anomalies, and geographical factors. Fetal echocardiography, with an overall sensitivity of 68.5% and a specificity of 99.8% [2], has been reported to play a critical role in this high-prevalence disease group by enabling early diagnosis, ensuring delivery of infants requiring intervention at appropriately equipped centers, and facilitating timely access to necessary medical care [3]. Given the critical role of fetal echocardiography in the early diagnosis and management of congenital heart disease, understanding the maternal and environmental risk factors that may contribute to its development is of substantial clinical importance.

Maternal smoking during the periconceptional period and throughout pregnancy may lead to the accumulation and systemic distribution of tobacco constituents in both the mother and the fetus, thereby posing significant risks across all stages of fetal development [4]. Exposure to tobacco smoke, particularly during early pregnancy, has been reported to adversely affect the development of vulnerable fetal structures and to suggest a potential teratogenic effect [5].

The literature includes several epidemiological and clinical studies reporting that maternal smoking during pregnancy may increase the overall risk of CHD and may exert more pronounced adverse effects on specific subgroups of cardiac defects [6,7,8,9]. These studies suggest that toxic constituents of tobacco smoke may influence fetal cardiovascular development through multifactorial mechanisms. However, in some investigations conducted within a similar scope, no significant association has been demonstrated between maternal smoking during pregnancy and the development of congenital heart disease, indicating the presence of inconsistent findings in the literature [10,11]. Moreover, data specifically focusing on populations referred for fetal echocardiography are limited. Such cohorts represent a clinically enriched group with a higher risk of cardiac anomalies and may provide additional insight into potential phenotype-specific associations that may not be fully captured in general-population-based studies, particularly in real-world clinical settings.

The aim of our study was to evaluate the association between maternal smoking during pregnancy and the risk of congenital heart disease using fetal echocardiographic data in a referred population. In addition, we aimed to examine the distribution of CHD subtypes according to maternal smoking status and to explore a potential dose–response relationship based on daily cigarette consumption.

## 2. Materials and Methods

### 2.1. Study Population

We conducted a retrospective review of fetal echocardiography reports from pregnant women referred to the pediatric cardiology clinic between 1 January 2024, and 1 November 2025, for evaluations performed at ≥20 weeks’ gestation. After applying predefined eligibility criteria, a total of 2715 pregnant women were included in the study, comprising 2463 non-smoking and 252 smoking pregnancies. Data extracted included maternal and paternal age, gestational age at examination, referring center and referral indication, maternal smoking status during pregnancy, fetal diagnoses, and gravida–parity–abortion–living counts.

Cases were excluded if a known chromosomal or syndromic abnormality was present; if the fetal echocardiographic assessment fell outside the study’s gestational-age window (evaluations not meeting the ≥20-week criterion); if the record represented a duplicate assessment from the same pregnancy (only the index examination retained); if the examination was performed off-site or under a nonstandard protocol precluding comparability; or if there was documented exposure to potent teratogens during pregnancy (e.g., isotretinoin, valproate, heavy alcohol use, laboratory-confirmed rubella or cytomegalovirus infection).

Daily cigarette consumption among smoking mothers was initially categorized into six groups: <1, 1–2, 3–4, 5–9, 10–19, and ≥20 cigarettes per day. For analytical purposes and to ensure adequate sample size within each stratum, these categories were consolidated, and participants were subsequently reclassified into four groups: non-smokers, <5 cigarettes/day, 5–9 cigarettes/day, and ≥10 cigarettes/day [12].

Institutional approval was obtained from the Ankara Etlik City Hospital Clinical Research Ethics Committee, and the study adhered to the principles of the Declaration of Helsinki.

### 2.2. Fetal Echocardiography

All measurements were conducted in line with the Practice Guideline for the Performance of Fetal Echocardiography issued by the American Institute of Ultrasound in Medicine (AIUM), developed in collaboration with the American College of Obstetricians and Gynecologists (ACOG), the Society for Maternal-Fetal Medicine (SMFM), and the American Society of Echocardiography (ASE), and endorsed by the American College of Radiology (ACR) [13]. Fetal echocardiograms were obtained by an experienced pediatric cardiologist using a GE Voluson S10 ultrasound system (GE Healthcare, Chicago, IL, USA) equipped with a high-frequency transducer (GE C1-5 RS, 1–5 MHz) and integrated software (version BT18).

### 2.3. Statistical Analysis

Descriptive data are reported as median (interquartile range) for continuous variables and as frequency (percentage) for categorical variables. The distribution of continuous variables was assessed using appropriate methods. For between-group comparisons, continuous variables were analyzed using the independent-samples *t* test or the Mann–Whitney U test, as appropriate, depending on data distribution, and categorical variables were compared using the χ^2^ test. To evaluate the association between smoking intensity and the presence of congenital heart disease, a binary logistic regression analysis was performed, with smoking intensity entered as an ordinal predictor to assess a linear trend. Odds ratios (ORs) with 95% confidence intervals (CIs) were calculated. All analyses were conducted in SPSS, version 25.0 (IBM Inc., Armonk, NY, USA). A two-sided type I error rate of 5% (*p* < 0.05) was considered statistically significant.

## 3. Results

A total of 2715 pregnant women who underwent fetal echocardiography after 20 weeks of gestation between 1 January 2024 and 1 November 2025 were included in the study. The records of 2784 fetuses were retrospectively analyzed. During the same study period, fetal echocardiographic findings of 2463 non-smoking pregnant women were compared with those of 252 women who continued smoking during pregnancy. Among the non-smoking pregnant women, 107 had discontinued smoking upon confirmation of pregnancy. In the non-smoking group, 2400 pregnancies were singleton, 59 were twin, and 4 were triplet pregnancies, yielding a total of 2530 fetuses. In the smoking group, 250 singleton and 2 twin pregnancies were identified, corresponding to 254 fetuses evaluated by fetal echocardiography (Table 1).

A statistically significant difference in maternal age was observed between the smoking and non-smoking groups (*p* = 0.025) (Table 2). As maternal age did not follow a normal distribution, intergroup comparisons were performed using the Mann–Whitney U test. Although the median maternal age was identical in both groups (29 years), the distributional characteristics differed markedly. Maternal ages in the non-smoking group were distributed across a wider range, reflecting a more heterogeneous pattern, whereas ages in the smoking group were clustered within a narrower interval. These findings indicate that, despite similar measures of central tendency, differences in distribution contributed to the observed statistical significance.

Fetal echocardiographic evaluation of the 254 fetuses in the smoking group revealed a broad spectrum of cardiac findings. The majority of cases (44%) demonstrated normal fetal echocardiographic results, followed by echogenic intracardiac focus (26.4%) and minimal tricuspid regurgitation (5.9%). Among structural cardiac anomalies, isolated ventricular septal defect (VSD) (5.9%) and tetralogy of Fallot (1.6%) were the most frequently identified conditions. The most common extracardiac findings were aberrant right subclavian artery (1.6%) and congenital diaphragmatic hernia (1.2%). A detailed distribution of all diagnoses is presented in Table 3.

A total of 2530 fetuses were evaluated in the non-smoking group and 254 fetuses in the smoking group. Congenital heart disease was identified in 316 fetuses (12.5%) in the non-smoking group and in 36 fetuses (14.2%) in the smoking group. Comparison of CHD prevalence between the two groups revealed no statistically significant difference (*p* = 0.442).

According to the classical morphological classification, the distribution of CHD subtypes did not differ significantly between the smoking and non-smoking groups (*p* = 0.607). Analysis of CHD subgroups within the study cohort demonstrated that septal defects were the most frequently identified diagnostic category in both groups, observed in 192 cases (60.8%) in the non-smoking group and 19 cases (52.8%) in the smoking group. Atrioventricular septal defects (AVSDs) were detected in 15 cases (4.7%) among non-smokers and in 1 case (2.8%) among smokers. Conotruncal defects were identified in 32 cases (10.1%) in the non-smoking group and in 7 cases (19.4%) in the smoking group. Left ventricular outflow tract (LVOT) obstructions were observed in 38 cases (12.0%) among non-smokers and in 5 cases (13.9%) among smokers. Right ventricular outflow tract (RVOT) obstructions were identified in 21 cases (6.6%) and 3 cases (8.3%), respectively. Heterotaxy was observed in 12 cases (3.8%) in the non-smoking group, whereas no cases were identified in the smoking group. Complex CHD was determined in 6 cases (1.9%) among non-smokers and in 1 case (2.8%) among smokers. Congenital heart defects were classified according to the classical morphological classification system, and distribution frequencies were compared between groups. A detailed distribution of all diagnoses and diagnostic subgroups according to maternal smoking status is presented in Table 4.

In the univariate analysis, maternal age (OR: 1.01, 95% CI: 0.98–1.02, *p* = 0.951) and smoking status (OR: 1.15, 95% CI: 0.79–1.67, *p* = 0.442) were not significantly associated with the risk of CHD, whereas diabetes mellitus (OR: 3.36, 95% CI: 1.97–5.73, *p* < 0.001), parental consanguinity (OR: 5.33, 95% CI: 3.43–8.23, *p* < 0.001), maternal febrile illness (OR: 2.37, 95% CI: 1.03–5.47, *p* = 0.041), maternal drug exposure (OR: 3.25, 95% CI: 2.44–4.33, *p* < 0.001), and a family history of CHD (OR: 2.49, 95% CI: 1.44–4.31, *p* = 0.001) were each significantly associated with an increased risk. In the multivariable analysis adjusting for potential confounders, parental consanguinity (OR: 4.58, 95% CI: 2.9–7.23, *p* < 0.001), maternal drug exposure (OR: 2.67, 95% CI: 1.96–3.63, *p* < 0.001), and family history of CHD (OR: 2.07, 95% CI: 1.16–3.68, *p* = 0.013) remained independently associated with an increased risk of CHD. Although diabetes mellitus showed a trend toward significance (OR: 1.75, 95% CI: 0.97–3.15, *p* = 0.06), it did not reach statistical significance after adjustment. Similarly, maternal febrile illness was no longer significantly associated with CHD (OR: 1.75, 95% CI: 0.93–4.02, *p* = 0.206). Importantly, no association was detected between maternal smoking and CHD after adjustment for confounders (OR: 0.92, 95% CI: 0.62–1.38, *p* = 0.708). These findings were consistent across sensitivity analyses. The detailed results of the univariate and multivariable analyses are presented in Table 5.

**Figure 1:**
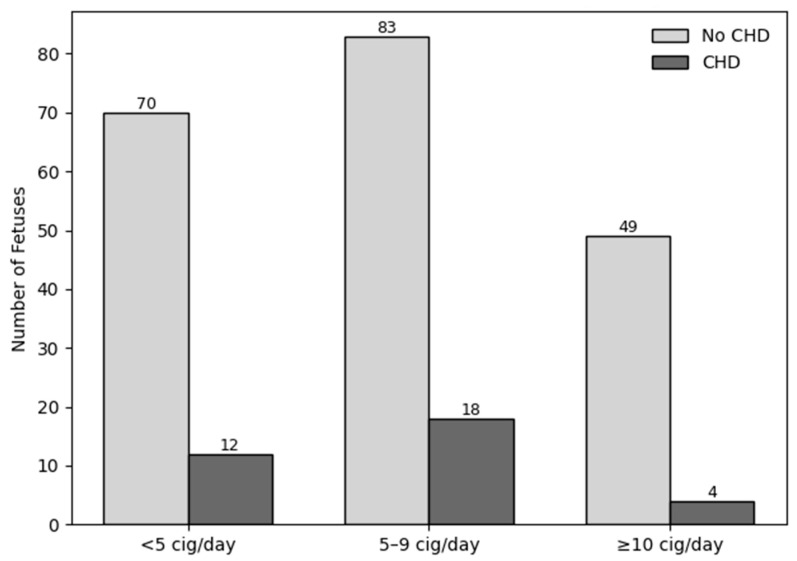
Distribution of CHD Within the Smoking Group According to Reclassified Maternal Daily Cigarette Consumption.

Daily cigarette consumption was reclassified into four exposure categories (non-smokers, <5 cigarettes/day, 5–9 cigarettes/day, and ≥10 cigarettes/day). Among 2530 non-smoking pregnancies, 316 fetuses were diagnosed with CHD, whereas CHD was identified in 12 of 82 pregnancies in the <5 cigarettes/day group, 18 of 101 in the 5–9 cigarettes/day group, and 4 of 53 in the ≥10 cigarettes/day group. The association between maternal smoking and CHD risk was evaluated using a logistic regression model. An association between smoking exposure and CHD was not detected (OR = 1.04; 95% CI: 0.86–1.26; *p* = 0.691). Although 254 fetuses were identified in the smoking group and 36 were diagnosed with CHD, the dose-based analysis included 236 fetuses and 34 CHD cases because daily cigarette consumption could not be specified for 18 pregnancies with reported smoking exposure; these cases were therefore excluded from the reclassified exposure analysis. The distribution of CHD across the reclassified smoking intensity categories within the smoking group and the corresponding regression results are presented in Figure 1.

## 4. Discussion

Maternal smoking has long been recognized as a significant public health concern due to its potential effects on fetal development. The particular susceptibility of embryonic cardiac development to environmental influences during early gestation has led to the hypothesis that maternal smoking may be associated with CHD. However, the strength and consistency of this association remain controversial, and the existing literature has not reached a clear consensus.

Previous studies have highlighted that variables such as maternal age and accompanying obstetric factors may influence the assessment of the association between maternal smoking and CHD. In our study, although a statistically significant difference in maternal age distribution was observed between the smoking and non-smoking groups, the identical median age in both groups and the fact that the difference primarily reflected distributional characteristics rather than central tendency suggest that this variable did not represent a clinically meaningful distinction between the groups.

Evaluation of the distribution of CHD subtypes indicates that VSD is the most frequently reported structural lesion in the general population, followed by atrial septal defect (ASD). Less common anomalies include AVSD, hypoplastic left heart syndrome, and conotruncal defects [14]. In our study, VSD was the most frequently identified structural anomaly in both the smoking and non-smoking groups, and the predominance of septal defects was consistent with the existing literature. Although ASD ranked second among septal defects, in line with previously reported epidemiological patterns, its overall frequency was lower than that of more complex lesions such as AVSD, hypoplastic left heart syndrome, and tetralogy of Fallot within the total distribution. Given that ASD is generally reported at higher frequencies in population-based data, this finding may be attributable to the referral pattern of our institution as a tertiary care center, where cases requiring advanced evaluation and specialized management are more likely to be referred. Accordingly, the relatively greater representation of complex and surgically significant cardiac anomalies in our study population may reflect the characteristics of a referral-based patient population.

In our study, evaluation of conotruncal defects revealed that tetralogy of Fallot appeared proportionally more frequent in the smoking group. Similarly, interrupted aortic arch cases were relatively more represented among fetuses exposed to maternal smoking. A prior investigation has suggested that high levels of occupational exposure to polycyclic aromatic hydrocarbons during early pregnancy may be associated with an increased risk of conotruncal heart defects, particularly tetralogy of Fallot [15]. Furthermore, parental smoking from one month before pregnancy through the first trimester has been implicated as a potential risk factor for conotruncal heart defects [16]. Although the proportional increase observed in conotruncal defects among the smoking-exposed group did not reach statistical significance, likely due to the limited number of cases within these subgroups, this observation should be interpreted with caution and considered exploratory.

Evaluation of left and right ventricular outflow tract obstructions, heterotaxy, and complex congenital heart disease subgroups revealed proportional differences across certain morphological subcategories between the two groups; however, these differences did not reach statistical significance. Examination of the distribution of CHD subtypes did not demonstrate a systematic or consistent shift toward any specific congenital heart defect in association with maternal smoking exposure. Collectively, these findings suggest that no association was detected between maternal smoking and a meaningful alteration in the distribution of congenital heart disease subtypes within the study population.

Benign follow-up findings such as minimal tricuspid regurgitation and echogenic intracardiac focus were frequently observed in our study, consistent with the existing literature. In particular, isolated mild tricuspid regurgitation in low-risk pregnancies has been reported to represent a predominantly physiological and transient finding, without an association with major structural cardiac anomalies in the postnatal period [17]. Similarly, the presence of an isolated echogenic intracardiac focus is not considered a strong predictor of chromosomal abnormalities or major structural cardiac pathology. Although associations with minor cardiac anomalies have been reported, this relationship appears to be more pronounced in right-sided foci that persist into the third trimester. In most instances, isolated echogenic intracardiac focus is widely accepted to follow a clinically benign course [18].

In this study, multivariable analysis demonstrated that parental consanguinity, maternal drug exposure during pregnancy, and a family history of congenital heart disease remained independently and significantly associated with an increased risk of CHD. In contrast, no independent association was detected between maternal smoking and CHD after adjustment for potential confounders. Although diabetes mellitus showed a strong association in the univariate analysis, it did not reach statistical significance in the multivariable model, remaining at a borderline level of significance (*p* = 0.06). Similarly, maternal febrile illness was significantly associated with CHD in the univariate analysis; however, this association did not persist after multivariable adjustment. These findings suggest that the observed associations of certain variables with CHD may be influenced by other clinical factors. The lack of a detected independent association between maternal smoking and CHD in this study is consistent with some previous reports [19,20], although the existing literature presents inconsistent findings [9,21,22]. This variability may be explained by differences in study populations, exposure definitions, and residual confounding. In contrast, the strong and independent associations observed for parental consanguinity, maternal drug exposure during pregnancy, and family history support the role of genetic susceptibility and intrauterine environmental factors in the pathogenesis of CHD. In line with these findings, prior studies have likewise demonstrated an increased risk of CHD among newborns of mothers with a family history of CHD, consanguineous marriages, maternal diabetes, antiepileptic drug use, and inadequate folic acid supplementation, and have also identified maternal co-morbidities, first-born status, and low birth weight as independent risk factors for CHD [20,23].

Following categorization of daily cigarette consumption into predefined exposure groups, the association between maternal smoking and the development of congenital heart disease was evaluated using a logistic regression model, and no statistically significant relationship was identified, nor was a consistent increase in CHD risk observed with increasing levels of cigarette consumption. However, the literature reports heterogeneous findings regarding the consistency and magnitude of the association between the amount of maternal smoking and CHD risk. In addition to meta-analyses reporting an increased risk of septal defects and atrioventricular septal defects associated with higher levels of maternal smoking [20,24], there are also studies indicating that heavy cigarette consumption (≥25 cigarettes/day) may be associated with right ventricular outflow tract obstructions when compared with fetuses of non-smoking mothers [9]. In a substantial proportion of these studies, the reported risk estimates were derived from subgroup analyses focusing on specific congenital heart defect subtypes. In our study, the limited number of cases within certain morphological categories did not allow for detailed subtype-specific analyses. Therefore, potential associations, particularly for rare lesions, may not have been fully captured. The distribution of smoking exposure in the study population was largely concentrated within the mild and moderate categories, whereas the heavy smoking group was relatively underrepresented. This imbalance may have constrained the ability to detect the magnitude of risk increases reported in the literature, particularly those observed at higher levels of cigarette consumption.

Compared to population-based studies, our referral-based fetal echocardiography cohort provides clinically enriched data from a high-risk population, offering complementary insights for prenatal evaluation and counseling in real-world settings.

### Limitations

Although the overall study population was large, the number of cases within certain CHD subgroups and higher smoking intensity categories was limited, which may have restricted the detection of subtle phenotype-specific associations and affected the stability of subgroup analyses. In addition, small subgroup counts in some morphological categories may have affected the stability and interpretability of subgroup comparisons. Furthermore, the single-center, observational design limits the generalizability of the findings and precludes causal inference. The absence of data on socioeconomic status and environmental exposures may have resulted in residual confounding, which should be considered when interpreting the findings. In addition, smoking status was based on maternal self-report without biochemical validation (e.g., cotinine levels), which may have introduced underreporting and misclassification, potentially biasing the results toward the null and masking a true association. Moreover, the inclusion of women who discontinued smoking after pregnancy confirmation in the non-smoking group may have introduced exposure misclassification, particularly for early pregnancy exposure. Furthermore, exclusion of cases with missing smoking intensity data in the dose–response analysis may have introduced bias within the smoking group. Finally, as the study population consisted exclusively of patients referred for fetal echocardiography, the cohort represents a higher-risk group enriched for suspected or known fetal abnormalities, which may not reflect the general obstetric population and may have influenced the observed associations by altering the baseline prevalence of congenital heart disease.

## 5. Conclusions

In this study population referred for fetal echocardiographic evaluation, no association was detected between maternal smoking during pregnancy and the risk of congenital heart disease or a meaningful alteration in CHD subtype distribution. In addition, no consistent increase in CHD risk was observed across categories of daily cigarette consumption. In this cohort, these findings suggest that no association was detected between maternal smoking exposure and the development or phenotypic pattern of CHD. Nevertheless, given the limited number of cases in specific morphological subgroups and higher smoking intensity categories, larger prospective investigations with sufficient subtype representation are warranted to further clarify potential phenotype-specific associations.

## Figures and Tables

**Table 1:** Distribution of Pregnancy Type and Number of Fetuses in the Study Population According to Maternal Smoking Status.

Pregnancy Characteristics	Study Population (*n*)	Non-Smoking Group (*n*)	Smoking Group (*n*)
Total pregnancies	2715	2463	252
Singleton pregnancies	2650	2400	250
Twin pregnancies	61	59	2
Triplet pregnancies	4	4	-
Total number of fetuses evaluated	2784	2530	254

**Table 2:** Comparison of Maternal Age According to Smoking Status.

Smoking Status	Minimum	Maximum	Median	*p*
Non-Smoking (*n* = 2463)	17	45	29	0.025
Smoking (*n* = 252)	18	48	29	

**Table 3:** Distribution of Fetal Echocardiographic Findings in Pregnancies with Maternal Smoking.

Diagnosis	*n* = 254	%
**Benign findings detected during follow-up**	205	80.7
Normal fetal echocardiographic findings	111	43.7
Echogenic intracardiac focus	67	26.4
Minimal TR	15	5.9
Echogenic intracardiac focus with minimal TR	10	3.9
Minimal MR	1	0.4
Septal hypertrophy	1	0.4
**CHD**	36	14.2
VSD	17	6.7
TOF	4	1.6
Interrupted aortic arch	2	0.8
ASD	2	0.8
Coarctation of the aorta	2	0.8
Isthmic and transverse aortic arch hypoplasia	2	0.8
Pulmonary atresia	2	0.8
Truncus arteriosus	1	0.4
Hypoplastic left heart syndrome (HLHS)	1	0.4
Complete AVSD	1	0.4
Tricuspid atresia	1	0.4
DIRV, malposition of the great arteries, and severe mitral stenosis	1	0.4
**Extracardiac Findings**	12	4.7
ARSA	4	1.6
Congenital diaphragmatic hernia with dextroposition	3	1.2
Single umbilical artery	1	0.4
Right aortic arch with right ductus arteriosus	1	0.4
Vein of Galen aneurysm	1	0.4
Hydrops fetalis with pericardial effusion	1	0.4
Isolated pericardial effusion	1	0.4
**Rhythm Findings**	1	0.4
Atrial extrasystole	1	0.4

Abbreviations: ASD: atrial septal defect; ARSA: aberrant right subclavian artery; AVSD: atrioventricular septal defect; CHD: congenital heart disease; DIRV: double-inlet right ventricle; MR: mitral regurgitation; TOF: tetralogy of Fallot; TR: tricuspid regurgitation; VSD: ventricular septal defect.

**Table 4:** Distribution of Pathologies Detected by Fetal Echocardiography According to the Classical Morphological Classification and Maternal Smoking Status.

		Non-Smoking Group (*n* = 316)	Smoking Group (*n* = 36)	*p*
		*n* (%)	*n* (%)	
Septal Defects	VSD	185 (96.4)	17 (89.5)	0.189
	ASD	7 (3.6)	2 (10.5)	
Atrioventricular Septal Defects	Complete AVSD	12 (80.0)	1 (100.0)	1.000
	İntermediate AVSD	2 (13.3)	0 (0.0)	
	Partial AVSD	1 (6.7)	0 (0.0)	
Conotruncal Defects	TOF	11 (34.4)	4 (80.0)	0.213
	Truncus Arteriosus	5 (15.6)	1 (20.0)	
	DORV	8 (25.0)	0 (0.0)	
	D-TGA	4 (12.5)	0 (0.0)	
	APW	2 (6.3)	0 (0.0)	
	Interrupted aortic arch	1 (3.1)	2 (28.6)	
	Hemitruncus	1 (3.1)	0 (0.0)	
Left Ventricular Outflow Tract Obstructions	HLHS	15 (39.5)	1 (20.0)	0.576
	Coarctation of the aorta	9 (23.7)	2 (40.0)	
	Isthmic and transverse aortic arch hypoplasia	6 (15.8)	2 (40.0)	
	Aortic stenosis	6 (15.8)	0 (0.0)	
	Coarctation of the aorta with aortic stenosis	2 (5.3)	0 (0.0)	
Right Ventricular Outflow Tract Obstructions	Tricuspid atresia	7 (33.3)	1 (33.3)	1.000
	Pulmonary atresia	9 (42.9)	2 (66.7)	
	Valvular PS	3 (14.3)	0 (0.0)	
	Ebstein anomaly	2 (9.5)	0 (0.0)	
Heterotaxy	LAI (Isolated)	2 (16.7)	0 (0.0)	-
	LAI + CHD	4 (33.3)	0 (0.0)	
	RAI + CHD	4 (33.3)	0 (0.0)	
	Situs inversus with dextrocardia	2 (16.7)	0 (0.0)	
Complex CHD	cc-TGA	3 (50.0)	0 (0.0)	0.143
	DILV	3 (50.0)	0 (0.0)	
	DIRV, malposition of the great arteries, and severe mitral stenosis	0 (0.0)	1 (100.0)	

Abbreviations: VSD, ventricular septal defect; ASD, atrial septal defect; AVSD, atrioventricular septal defect; TOF, tetralogy of Fallot; DORV, double-outlet right ventricle; D-TGA, dextro-transposition of the great arteries; APW, aortopulmonary window; HLHS, hypoplastic left heart syndrome; PS, pulmonary stenosis; LAI, left atrial isomerism; RAI, right atrial isomerism; CHD, congenital heart disease; cc-TGA, congenitally corrected transposition of the great arteries; DILV, double-inlet left ventricle; DIRV, double-inlet right ventricle.

**Table 5:** Univariate and Multivariate Analyses for Congenital Heart Disease.

	Univariate Analysis		Multivariate Analysis	
	OR (95% CI)	*p*	OR (95% CI)	*p*
Age	1.01 (0.98–1.02)	0.951		
Diabetes Mellitus * (Present vs. Absent)	3.36 (1.97–5.73)	**<0.001**	1.75 (0.97–3.15)	0.06
Parental Consanguinity (Present vs. Absent)	5.33 (3.43–8.23)	**<0.001**	4.58 (2.9–7.23)	**<0.001**
Maternal Febrile Illness (Present vs. Absent)	2.37 (1.03–5.47)	**0.041**	1.75 (0.93–4.02)	0.206
Maternal Drug Exposure (Present vs. Absent)	3.25 (2.44–4.33)	**<0.001**	2.67 (1.96–3.63)	**<0.001**
Family History of Congenital Heart Disease (Present vs. Absent)	2.49 (1.44–4.31)	**0.001**	2.07 (1.16–3.68)	**0.013**
Smoking Status (Smoking vs. Non-smoking)	1.15 (0.79–1.67)	0.442	0.92 (0.62–1.38)	0.708

CI: confidence interval, OR: odds ratio * Including type 1, type 2 and gestational diabetes mellitus.

## Data Availability

The data that support the findings of this study are available from the corresponding author upon reasonable request.
